# Supplementary material for: The relationship between psychological characteristics of patients and their utilization of psychiatric inpatient treatment: A cross-sectional study, using machine learning
Source: PLoS One. 2022 Apr 1;17(4):e0266352. doi: 10.1371/journal.pone.0266352 (PMC8975161; doi:10.1371/journal.pone.0266352)
Supplement: S1 Table — (DOCX) [file pone.0266352.s001.docx]

| **S1 Table.** Correlation matrix containing bivariate correlations between all relevant variables | | | | | | | | | | | | | | | | | | | | | | |
| --- | --- | --- | --- | --- | --- | --- | --- | --- | --- | --- | --- | --- | --- | --- | --- | --- | --- | --- | --- | --- | --- | --- |
| **Variable** | **1** | **2** | **3** | **4** | **5** | **6** | **7** | **8** | **9** | **10** | **11** | **12** | **13** | **14** | **15** | **16** | **17** | **18** | **19** | **20** | **21** | **22** |
| 1. Utilization pattern | 1 |  |  |  |  |  |  |  |  |  |  |  |  |  |  |  |  |  |  |  |  |  |
| 2. Length of stay | 0.66 | 1 |  |  |  |  |  |  |  |  |  |  |  |  |  |  |  |  |  |  |  |  |
| 3. Number of admissions | 0.83 | 0.74 | 1 |  |  |  |  |  |  |  |  |  |  |  |  |  |  |  |  |  |  |  |
| 4. Sex | -0.01 | -0.04 | 0.01 | 1 |  |  |  |  |  |  |  |  |  |  |  |  |  |  |  |  |  |  |
| 5. Age | 0.13 | 0.05 | 0.13 | 0.16 | 1 |  |  |  |  |  |  |  |  |  |  |  |  |  |  |  |  |  |
| 6. Psychopathology | 0.18 | 0.19 | 0.15 | 0.11 | 0.09 | 1 |  |  |  |  |  |  |  |  |  |  |  |  |  |  |  |  |
| Quality of life |  |  |  |  |  |  |  |  |  |  |  |  |  |  |  |  |  |  |  |  |  |  |
| 7. Physical health | 0.01 | -0.05 | 0.06 | 0.01 | 0.01 | -0.26 | 1 |  |  |  |  |  |  |  |  |  |  |  |  |  |  |  |
| 8. Psychological health | 0.03 | 0.00 | 0.11 | 0.09 | 0.08 | -0.26 | 0.70 | 1 |  |  |  |  |  |  |  |  |  |  |  |  |  |  |
| 9. Social relationships | -0.07 | -0.05 | -0.01 | 0.20 | 0.10 | -0.18 | 0.37 | 0.57 | 1 |  |  |  |  |  |  |  |  |  |  |  |  |  |
| 10. Environment | -0.06 | -0.13 | -0.01 | 0.06 | 0.10 | -0.28 | 0.52 | 0.63 | 0.47 | 1 |  |  |  |  |  |  |  |  |  |  |  |  |
| 11 Self-esteem | -0.13 | -0.09 | -0.05 | 0.07 | -0.02 | -0.22 | 0.51 | 0.81 | 0.51 | 0.52 | 1 |  |  |  |  |  |  |  |  |  |  |  |
| Self-stigma |  |  |  |  |  |  |  |  |  |  |  |  |  |  |  |  |  |  |  |  |  |  |
| 12. Awareness | 0.12 | 0.03 | 0.05 | 0.02 | -0.02 | -0.14 | -0.14 | -0.13 | -0.12 | -0.16 | -0.10 | 1 |  |  |  |  |  |  |  |  |  |  |
| 13. Agreement | 0.04 | 0.15 | 0.00 | -0.07 | 0.06 | 0.02 | -0.14 | -0.16 | -0.10 | -0.27 | -0.16 | 0.39 | 1 |  |  |  |  |  |  |  |  |  |
| 14. Application | 0.01 | 0.03 | -0.04 | -0.27 | -0.15 | 0.21 | -0.43 | -0.54 | -0.33 | -0.36 | -0.50 | 0.14 | 0.35 | 1 |  |  |  |  |  |  |  |  |
| 15. Hurts-self | 0.15 | 0.18 | 0.10 | -0.09 | -0.15 | 0.19 | -0.54 | -0.68 | -0.39 | -0.45 | -0.66 | 0.15 | 0.26 | 0.69 | 1 |  |  |  |  |  |  |  |
| Subjective experience and meaning of psychoses |  |  |  |  |  |  |  |  |  |  |  |  |  |  |  |  |  |  |  |  |  |  |
| 16. Biographical integration | 0.10 | 0.04 | 0.07 | -0.07 | -0.10 | -0.14 | -0.01 | -0.03 | -0.05 | -0.16 | 0.05 | 0.15 | 0.06 | 0.03 | 0.11 | 1 |  |  |  |  |  |  |
| 17. Symptoms positive | 0.06 | 0.12 | 0.09 | -0.15 | -0.18 | -0.20 | 0.07 | 0.13 | 0.17 | 0.00 | 0.26 | 0.13 | 0.04 | -0.03 | 0.02 | 0.48 | 1 |  |  |  |  |  |
| 18. Symptoms negative | 0.03 | -0.06 | -0.11 | -0.01 | -0.02 | 0.12 | -0.39 | -0.44 | -0.39 | -0.29 | -0.42 | -0.08 | 0.04 | 0.27 | 0.41 | -0.07 | -0.33 | 1 |  |  |  |  |
| 19. Positive consequences | -0.08 | 0.01 | -0.03 | -0.23 | -0.27 | -0.18 | 0.19 | 0.20 | 0.13 | 0.10 | 0.33 | 0.08 | -0.04 | -0.10 | -0.11 | 0.39 | 0.61 | -0.22 | 1 |  |  |  |
| 20. Negative consequences | 0.03 | 0.06 | 0.02 | -0.07 | -0.19 | 0.09 | -0.41 | -0.52 | -0.36 | -0.35 | -0.43 | 0.18 | 0.12 | 0.40 | 0.54 | 0.15 | 0.20 | 0.42 | 0.12 | 1 |  |  |
| Insight into the disease |  |  |  |  |  |  |  |  |  |  |  |  |  |  |  |  |  |  |  |  |  |  |
| 21. Self-reflectiveness | -0.04 | -0.11 | -0.08 | -0.19 | -0.30 | -0.13 | -0.22 | -0.29 | -0.20 | -0.13 | -0.25 | 0.09 | 0.09 | 0.27 | 0.37 | 0.18 | 0.12 | 0.31 | 0.19 | 0.35 | 1 |  |
| 22. Self-certainty | 0.06 | 0.15 | -0.01 | -0.07 | -0.02 | 0.11 | 0.21 | 0.20 | 0.05 | 0.06 | 0.17 | -0.08 | 0.00 | -0.18 | -0.18 | 0.00 | -0.02 | -0.03 | 0.08 | -0.06 | -0.36 | 1 |

*Note.* Cronbach’s alphas are shown in the diagonal.
